# Mechanistic Insights into the Reaction of Chlorination of Tryptophan Catalyzed by Tryptophan 7-Halogenase

**DOI:** 10.1038/s41598-017-17789-x

**Published:** 2017-12-12

**Authors:** Tatyana G. Karabencheva-Christova, Juan Torras, Adrian J. Mulholland, Alessio Lodola, Christo Z. Christov

**Affiliations:** 1 Department of Chemistry, Michigan Technological University, Houghton, 49931 MI USA; 2 Department of Chemical Engineering, Escola d’Enginyeria de Barcelona Est (EEBE), Universitat Politècnica de Catalunya, C. Eduard Maristany 10-14, 08019 Barcelona, Spain; 3 Centre for Computational Chemistry, School of Chemistry, University of Bristol, Cantock’s Close, Bristol, BS8 1TS UK; 4 Pharmacy Department, Università di Parma, V. le P.G Usberti 27/A, Campus Universitario, 431124 Parma, Italy

## Abstract

Tryptophan 7-halogenase catalyzes chlorination of free tryptophan to 7-chlorotryptophan, which is the first step in the antibiotic pyrrolnitrin biosynthesis. Many biologically and pharmaceutically active natural products contain chlorine and thus, an understanding of the mechanism of its introduction into organic molecules is important. Whilst enzyme-catalyzed chlorination is accomplished with ease, it remains a difficult task for the chemists. Therefore, utilizing enzymes in the synthesis of chlorinated organic compounds is important, and providing atomistic mechanistic insights about the reaction mechanism of tryptophan 7-halogenase is vital and timely. In this work, we examined a mechanism for the reaction of tryptophan chlorination, performed by tryptophan 7-halogenase, by calculating potential energy and free energy surfaces using two different Combined Quantum Mechanical/Molecular Mechanical (QM/MM) methods both employing Density Functional Theory (DFT) for the QM region. Both computational strategies agree on the nature of the rate-limiting step and provided close results for the reaction barriers of the two reaction steps. The calculations for both the potential energy and the free energy profiles showed very similar geometric features and hydrogen bonding interactions for the characterized stationary points.

## Introduction

**Figure 1:**
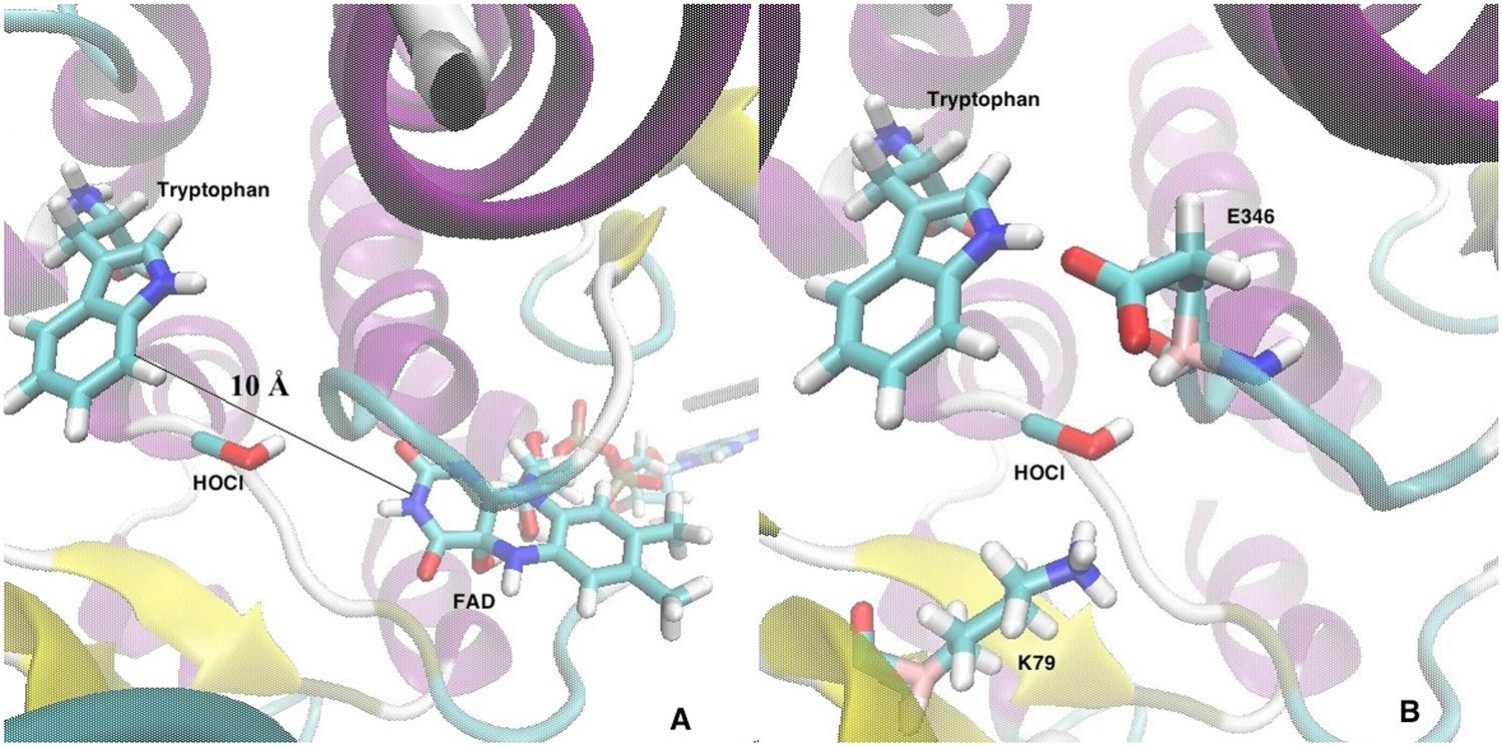
(**A**) Modified X-ray crystallographic structure of tryptophan 7-halogenase, shown in new cartoon representation. Line-represents the 10 Å distance between FAD (cofactor) binding site and tryptophan (substrate) binding site, HOCl (chlorinating agent) shown in licorice representation; carbon atoms - in cyan, nitrogen atoms - in blue, oxygen atoms - in red, and hydrogen atoms - in white. 1 (**B**) Zoom in view of the active site, representing the main actors of catalysis - the substrate tryptophan, the chlorinating agent - HOCl and K79 and E346 residues. Coordinates taken from PDB entry 2AR8.

**Figure 2:**
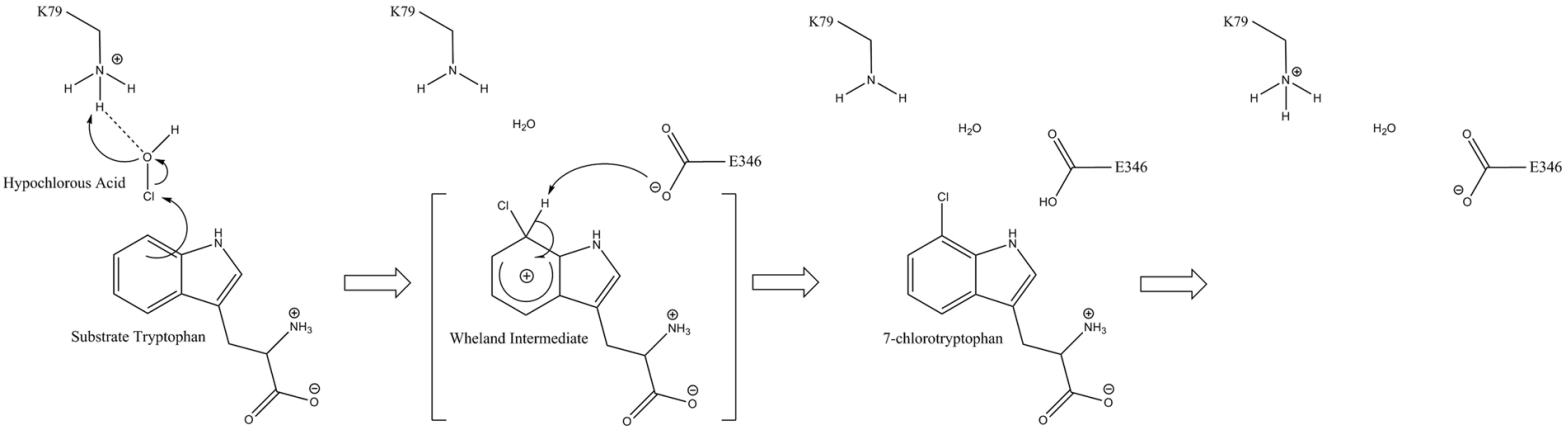
Proposed reaction mechanism of chlorination of tryptophan catalyzed by tryptophan 7-halogenase. First reaction step - formation of an arenium ion (Wheland intermediate) and second reaction step - deprotonation of the Wheland intermediate by the E346 oxygen. At the final stage of the mechanism K79 and E346 are regenerated and ready for a new enzymatic cycle of the reaction. A hydrogen bond interaction between K79 hydrogen and HOCl oxygen is denoted by a dashed line.

Halogenation reactions play an important role in the living systems^1–3^. More than five thousand halogenated natural compounds have been synthesized in organisms such as bacteria, fungi, plants, and mammals^1–3^. Introduction of a halogen atom can dramatically increase the biological function of the modified compound, for example incorporation of two chlorine atoms in the antibiotic vancomycin is critical for its antibacterial activity^1–3^. Chlorination of free tryptophan to 7-chlorotryptophan by enzyme tryptophan 7-halogenases is the first step in the biosynthesis of pyrrolnitrin and rebeccamycin. Based on information obtained from structural studies, a reaction mechanism for the enzymatic chlorination of tryptophan was proposed. It was suggested that in the catalytic cycle of tryptophan 7-halogenase the reduced form of its cofactor - flavin adenine dinucleotide (FAD) - FADH_2_ first reacts with molecular oxygen O_2_ to C4a-peroxyflavin, which then reacts with chlorine forming hypochlorous acid (HOCl). The crystal structure of tryptophan 7-halogenase reveals that tryptophan—substrate, and FAD—cofactor, are located in different binding sites and are separated by 10 Å distance^4^ (Fig. 1A). Therefore, HOCl formed in the FAD binding site moves through a tunnel in the enzyme and becomes activated to chlorinate the substrate^4^ in the tryptophan binding site. A hydrogen bonding interaction between HOCl and lysine 79 subsequently activates HOCl by increasing its electrophilicity, which facilitates the chlorination reaction. Tryptophan halogenation in solution produces a mixture of halogenated tryptophan products, therefore it lacks regioselectivity^5^. The regioselectivity in tryptophan 7-halogenase might be achieved via active site interactions mutually orienting the substrate tryptophan C7 atom and the chlorinating agent HOCl appropriately for chlorination. In the tryptophan binding site the reaction of chlorination of tryptophan proceeds as an aromatic electrophilic substitution taking place in two distinct chemical steps (Fig. 2): (1) insertion of a chlorine atom with the formation of an arenium ion/σ-complex (Wheland intermediate), and (2) proton removal of this reactive intermediate by a nearby glutamate 346 (E346), leading to formation of the product 7-chlorotryptophan^4^. The following experimental results confirm the role of both residues as being essential for catalysis - the E346Q mutation significantly affects the reaction (slowing it down by two orders of magnitude) and the K79A mutation stops it completely^4^. The electrophilic attack on the tryptophan substrate can be carried out via two possible mechanisms: (1) direct attack by activated HOCl or (2) an attack by a preliminarily formed lysine 79 bound chloramine^6^ (formed by reacting with HOCl). However, chloramines are milder chlorinating agents than HOCl, more selective toward their substrate and therefore, not sufficient for chlorination^7^. Quantum mechanical (QM) calculations of N-chloramine, showed reduced charge on the Cl atom of HOCl upon N-chloramine formation, and hence reduced electrophilicity compared to free HOCl^7^. In addition, the N-chloramine proposal for being the chlorinating agent in the enzyme reaction mechanism is based on experimental investigation of a different tryptophan 7-halogenase called rebeccamycin halogenase (RebH) with known crystal structure^8^ which shares 55% sequence identity with tryptophan 7-halogenase and catalyzes the same overall reaction of chlorination of tryptophan at C7 atom, but as a part of the biosynthetic pathway of rebeccamycin. Our modelling studies are focused on the mechanism of direct attack by HOCl, a proposal based on examination of tryptophan 7-halogenase structure. The enzymatic chlorination of tryptophan is a highly regioselective process^3^ facilitated by well-designed interactions between the chlorinating agent and key catalytic active site residues^4,7^.

Experimental kinetic data (k_cat_ values) were measured for the whole enzymatic cycle of tryptophan 7-halogenase that includes: reduction of the cofactor FAD, formation of FAD-peroxide, formation of HOCl, and chlorination of tryptophan by HOCl. However, no experimental data (kcat values) were measured specifically for the reaction of chlorination of tryptophan (the reaction mechanism modelled by us in this paper). Additionally, there is no experimental data showing which step in the whole cycle is the rate-limiting one.

Despite that a large amount of experimental structural information and kinetic data has been accumulated^4,7^, mechanistic details and energetics of the reaction of tryptophan chlorination, by tryptophan 7-halogenase, that can be revealed computationally, remain unknown. To the best of our knowledge there are no published computational modelling studies on the reaction mechanism of tryptophan 7-halogenase. The activation barriers, the structures of the transition states for the two chemical steps and the key interactions between reactants, transition states, products and the protein are not known. It is vitally important to determine the atomistic mechanism of enzymatic regioselective halogenation of tryptophan because many biologically and pharmaceutically active natural products contain a chlorine atom. In addition, utilizing enzymes in the synthesis of chlorinated organic compounds requires detailed understanding of the reaction mechanism of enzymatic chlorination. Furthermore, the enzymatic chlorination performed by tryptophan 7-halogenase, makes it an attractive subject as a biocatalyst for a potential biotechnological application.

Combined Quantum Mechanical/Molecular Mechanical (QM/MM) methods have been applied for investigating enzyme catalyzed reaction mechanisms^9,10^. A comparative study of different QM/MM methods for understanding of enzyme catalyzed reaction mechanism at ab initio, DFT and semiempirical levels was recently published^11^. By comparing potential energy and free energy profiles of the simulated reaction, an estimate for the activation entropy can be made. In general, performing QM/MM free energy reaction path calculations at density functional theory (DFT) level is still challenging due to the significant computational time required for DFT calculations, but also due to the large number of calculations necessary to ensure proper sampling of the conformational space of reactants, transition states and products^12^. However, the development of new tools and methodologies allowed for increased number of QM/MM reaction path free energy studies at ab initio and DFT level of theory for the QM region^13^. Recently, a multiscale study combining hybrid QM/MM-MD, multiple steering MD, and the Minh-Adib estimator reproduced experimental thermodynamics data of Diels-Alder reaction, the activation free energy barrier, and the effect of different solvent environments such as water and methanol^14^ was revealed.

To complement the missing, but vitally important, knowledge about the reaction mechanism of the tryptophan 7-halogenase, we applied two different computational approaches in studying the two chemical steps of the enzymatic chlorination of tryptophan. In particular, we focused on QM/MM potential energy and QM/MM free energy reaction paths of the most important part of the catalytic cycle of the proposed reaction mechanism of tryptophan 7-halogenase - the process of tryptophan chlorination by HOCl. We applied two different QM/MM methods: one to reveal the potential energy reaction path, and another to gain insight about the free energy changes along the reaction; both of them applying Density Functional Theory level for the quantum part of the system. Experimental data suggests that HOCl is formed, and then migrates from its site of formation in the FAD binding site to the substrate’s tryptophan binding site where the reaction of chlorination takes place^4^. Exploration of potential energy and free energy profiles were carried out by means of QM/MM potential energy reaction path approach at B3LYP/6–31 + G(d)-CHARMM27 level, and Multiple Steering Molecular Dynamics method using the QM/MM-MD approach through Amber-PUPIL-NWChem (B3LYP/6–31 G) interface^15,16^, respectively. Here, we explored the common trends and specificities for the potential and free energy reaction profiles, the structures of the stationary points and revealed key interactions between the reactants and the enzyme active site, which may contribute to the catalytic efficiency of the process.

## Methods

### Choice of X-ray crystal structure for modelling

A number of X-ray crystallographic structures of tryptophan 7-halogenase are available in the Protein Data Bank (PDB)^17^. The X-ray crystallographic structure with PDB ID: 2AR8^4^ isolated from *Pseudomonas fluorescens* with a resolution of 2.20 Å, was chosen for modelling. The structure contains the following components - a chloride ion (Cl^−^), a flavin adenine dinucleotide (FAD) cofactor and the reaction product, 7-chlorotryptophan. This structure was chosen for modelling because it is the only structure that contains 7-chlorotryptophan, which was used to model the Reactant Complex (RC). In order to reproduce the reactant complex structure, the product 7-chlorotryptophan was modified to the substrate tryptophan and HOCl.

Variations in the system setup depended on the particular simulation type applied to the system, namely: potential energy path or free energy path modelling, and are detailed in the supporting information (SI).

### QM/MM Potential Energy Reaction Path Calculations

In all QM/MM calculations the protein was ‘divided’ into two parts - a quantum mechanical (QM) part and a molecular mechanical (MM) part. The quantum mechanical (QM) part consisted of HOCl, the substrate tryptophan, and the side chains of lysine 79 (K79) and glutamate 346 (E346) (Figure S1 in SI), that are proposed to play key roles in the reaction mechanism^4,7–11^ of the enzyme. The molecular mechanical (MM) part involved the rest of the system. This resulted in 57 atoms in total for the quantum mechanical region which had a net charge of zero. Most QM/MM systems have the QM and MM regions in the same molecule and thus, it is necessary to “cut” at least one covalent bond to define the two regions. In cases like this it is necessary to fill the unsaturated valence of the QM atom. Therefore, for our system of interest at the points where the QM/MM boundary bisects a covalent bond, the valences of the QM atoms were saturated using the link-pair formalism^18^. Two hydrogen link atoms were added to the side chains of E346 and K79, respectively.

**Figure 3:**
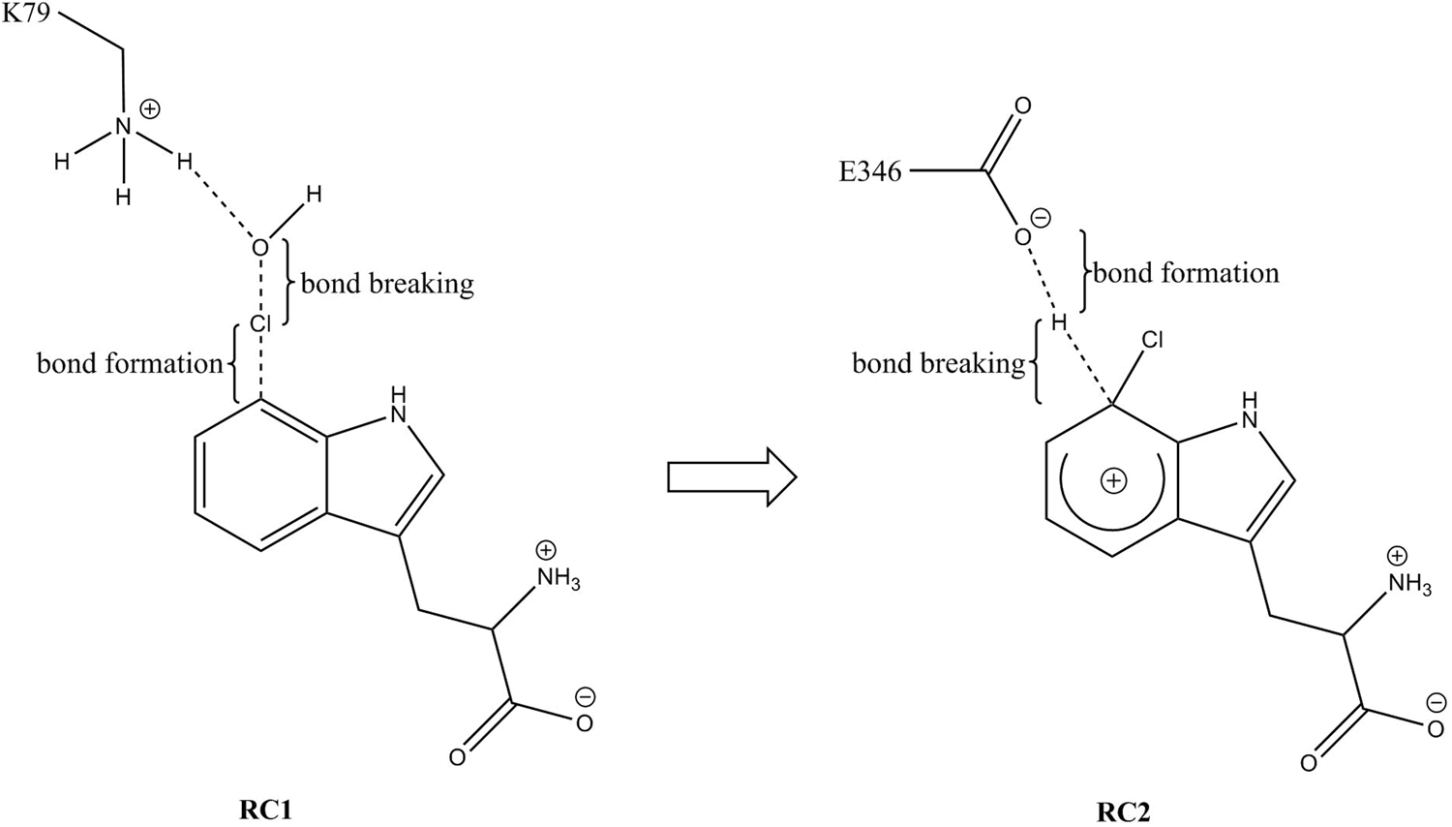
Schematic representation of reaction coordinate RC1 on the left and reaction coordinate RC2 on the right. RC1 is describing the process of bond breaking between O and Cl atoms of HOCl, and bond formation between the Cl atom of HOCl and C7 atom from the indole ring of tryptophan. RC2 is defined as a linear combination of the distance between the C7 atom of the Wheland intermediate and its H atom, that breaks, and the distance between the Wheland intermediate H atom and the O atom of the carboxylate group E346 that forms.

Potential energy reaction path modelling was performed with the adiabatic mapping method^19,20^ at B3LYP/6–31 + G(d)-CHARMM27 using the QoMMMA^21^ interface. The adiabatic mapping method has been shown to elucidate the reaction mechanism of many enzymes including FAAH^22^, COX-1^23^ and chorismate mutase^24^. The B3LYP functional has been largely applied for studying aromatic halogenation^25,26^ and in large number of QM/MM studies of enzyme-catalyzed reaction mechanisms^9,23,24,27^. The QM calculations were performed using the Jaguar software^28^ and the MM atoms in the system were treated by CHARMM27 all-atom force field^29^ implemented in the Tinker MM code^30,31^. The MM atoms were optimized during each step of the QM optimization and the polarization effects were accounted for by including the point charges of all MM atoms in the QM Hamiltonian. The transition states were determined approximately, as the highest energy points along the respective reaction profile. The reaction coordinate for the formation of the Wheland intermediate (RC1, Fig. 3) was chosen as: bond breaking between the O and Cl atoms of HOCl, and the formation of a bond between the Cl atom of HOCl and the 7^th^ C atom from the indole ring of tryptophan: RC1 = d_1_[O-Cl] − d_2_[Cl-C].

The reaction coordinate used for describing the proton abstraction from the Wheland intermediate by the oxygen of E346 (RC2, Fig. 3) was defined as a linear combination of the following distances: the distance between the 7^th^ C atom of the Wheland intermediate and its hydrogen which describes bond breaking, and the distance between the Wheland intermediate hydrogen and the E346 oxygen of its carboxylate group, which describes the formation of a bond, RC2 = d_1_[C-H] − d_2_[H-O].

### QM/MM-MD Free Energy Reaction Path Calculations

The free energy reaction profile along the respective reaction of interest was obtained by statistical treatment of the work function, along the chosen reaction coordinate, using different trajectories via the Multiple Steered Molecular Dynamics (MSMD) method, which relates the system’s non-equilibrium dynamics to its equilibrium properties^32,33^. The MSMD method considers a system as a subject to external time-dependent perturbation [*λ* = *λ(t)*], described by the Hamiltonian *H (* ***r***, *λ)*. Where *∆G(λ)* is the free energy change and *W(λ)* is the external work performed on the system, as it evolves from an initial to a final state (*λ*_0_ *→* *λ*). The Jarzynski relationship^34^ and the Crooks fluctuation theorem^35^ allow for relating the non-equilibrium work distribution and the equilibrium free energy differences. This allows for recovery of arbitrary equilibrium ensemble averages from measurements of driven non-equilibrium processes.

In particular, the Crooks’s path-ensemble average theorem^36^ relates the forward average of an arbitrary functional **F**(λ) of the phase space trajectory, to its work-weighted average in the reverse process,

1 $${\langle F\rangle }_{F}={\langle \hat{F}{e}^{-\beta (W-\Delta F)}\rangle }_{R}$$

The forward average <···>_F,_ is an average over all trajectories (path-ensemble average) generated in the forward process, wherein the external parameter *λ*, is driving the system from the initial to the final state (e.g., reactants to products in a pulling experiment). Similarly, <···>_R_ defines the average in the reverse direction. *∆F* is the free energy difference between the equilibrium states, corresponding to the final and initial points. This result offers a means of including of the reverse process in the forward path ensemble, when their arbitrary functional is reweighted by **e** ^*β*(*W*−Δ*F*)^. Based on those theorems, Minh and Adib^37^, proposed a bidirectional PMF estimator that includes trajectories from the forward and reverse perturbation along the reaction coordinate,

2 $${e}^{-\beta {G}_{0}(\lambda )}=\frac{{\sum }_{t}[ < \frac{{n}_{F}\delta (\lambda -{\lambda }_{t}){e}^{-\beta {W}_{0}^{t}}}{{n}_{F}+{n}_{R}{e}^{-\beta (W-\Delta F)}}{ > }_{{F}}+ < \frac{{n}_{R}\delta (\lambda -{\lambda }_{\tau -t}){e}^{-\beta {W}_{\tau -t}^{\tau }}}{{n}_{F}+{n}_{R}{e}^{\beta (W+\Delta F)}}{ > }_{{R}}]{\in }}{{\sum }_{t}{e}^{-\beta [V(\lambda ;t)-\Delta {F}_{t}]}}$$

where G_0_(λ) is the potential of mean force of the unperturbed system and *∆F*_*t*_ *=* *F*_*t*_ *−* *F*_0_ is the free energy difference between the equilibrium states defined at time t and 0, respectively. V(λ;t) is the time-dependent potential acting on a collective coordinate (e.g., harmonic potential along the reaction coordinate). The Mihn-Adib PMF estimator has been used previously in several works such as reconstructing free energy path of DNA stretching experiments^38^ and describing RNA/peptide binding/unbinding processes^39^.

The use of Jarzynski’s equality was validated on several systems, including deca-alanine stretching^40,41^, most of them involving classical MD. The methodology has been compared to existing biased MD techniques such as umbrella sampling^42^ and targeted MD^43^, and the results were comparable. Potential energy path simulations have been widely applied by means of hybrid QM/MM approach using semi-empirical methods (e.g. PM3, AM1)^44^. However, a few potential of mean force calculations have been performed using DFT QM/MM-MD methods in large systems for simulating processes such as unfolding and ligand diffusion in proteins^45^, reaction mechanism involving small molecules^46–48^ and some specific chemical reactions of biochemical interest^49^ because of their computational cost. Steered-MD and QM/MM calculations have been used to elucidate enzymatic reactions^50^ including covalent enzyme-inhibitor reactions^51^.

The free energy reaction profiles were generated by applying hybrid quantum mechanical/molecular mechanical - molecular dynamics (QM/MM-MD) calculations conducted by the AMBER^52^-PUPIL^53^-NWChem^54^ interface^15,16^ and ff03.r1^55,56^ Amber force field, used to describe the MM part of the simulated system. The B3LYP/6–31 G functional was used to describe the quantum mechanical (QM) part of the system. Specific parameters for the cofactor FAD were obtained from the R.E.D. project^57^. The QM part of the simulated system chosen for the free energy path scanning was very similar to the one applied in the potential energy reaction path simulations; it includes HOCl and the side chain of K79. The only difference was that it also included the whole E346 residue and only the side chain of the substrate tryptophan (Figure S2 in the SI). The link-pair formalism was used to treat the covalent bonds that cross the QM/MM boundary and thus, four hydrogen link atoms were added to tryptophan, K79, L345, and S347, respectively. A classical MD simulation was performed for 5 ns and fifteen different structures were extracted from the classical production trajectory, as initial structures, for generating the free energy paths (see SI). All the fifteen initial structures were relaxed under the hybrid QM/MM-MD approach during 0.5 ps (1000 steps), before pulling through the reaction coordinate. Distance constraints were applied to HOCl, K79 and tryptophan. For each simulation, a harmonic potential with a force constant of 5000 kcal mol^−1^ Å^−2^ was applied. The center of the potential was moved along the reaction coordinate during all 3000 steps of the MD simulation. The step size was 0.5 fs. A reverse reaction was simulated for the fifteen final structures obtained by the pulling from reactants to products. Free energy paths were studied using two different reaction coordinates (RC) depending on the reaction step namely: the RC1 reaction coordinate was used for simulating the reaction of formation of the Wheland intermediate and the RC2 reaction coordinate was used for simulating its consequent deprotonation. RC1 was defined as a lineal combination of distances involved in the formation of two bonds - namely the bond between the hydrogen atom of K79 and the oxygen atom of HOCl, and the bond between the chlorine atom of HOCl, and the 7^th^ carbon atom of the indole ring of tryptophan: RC1 *=* −d_1_[H-O] − d_2_[Cl-C].

**Figure 4:**
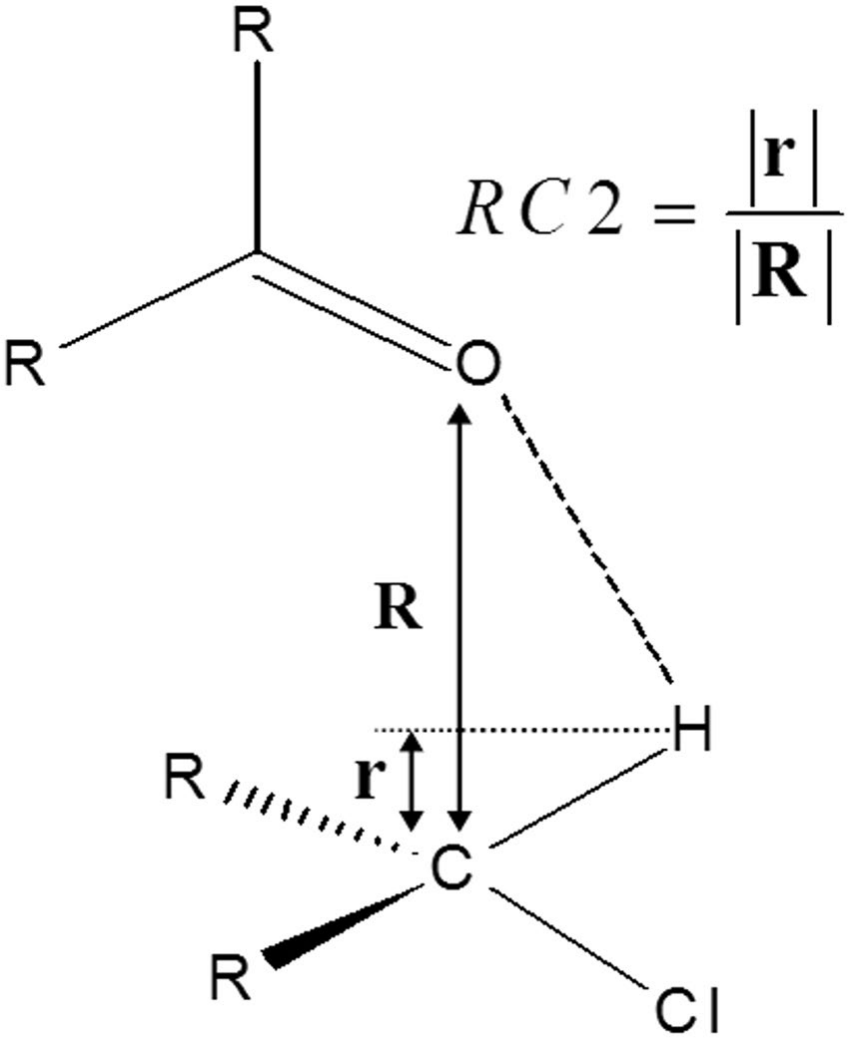
Schematic representation of the mid-plane reaction coordinate, RC2, applied for free energy reaction profile calculation of the second step of the reaction mechanism of chlorination of tryptophan – deprotonation of the Wheland intermediate by E346.Where C is the Wheland intermediate carbon, and O is the E346 oxygen atom, which accepts the proton. The mid-plane RC2 is defined as the ratio r/R, where R is the distance between C and O, and r is the length of the projection of the C-H distance onto distance R.

For modelling the reaction of deprotonation of the Wheland intermediate, a reaction coordinate that describes the formation of a bond between the oxygen atom of the carboxyl group of E346 and the hydrogen atom of the Wheland intermediate was chosen. In this process, which involves transfer of a proton from the Wheland intermediate carbon to E346 oxygen, a mid-plane reaction coordinate was used (RC2, Fig. 4). Taking into account that C is the Wheland intermediate carbon, to which the hydrogen atom is initially bound to, and O is the E346 oxygen atom, to which the proton will be transferred to, the mid-plane reaction coordinate, RC2 was defined as the ratio r/R, where R is the length of the distance between C and O and r is the length of the projection of the C-H distance onto distance R. RC2 reaction coordinate is dimensionless since it is a ratio between two distances. Mid-plane reaction coordinate is one of the most appropriate approaches for reproducing reversible pathways in studying proton transfer processes, and is the least susceptible one to hysteresis^58^.

The free energy reaction profiles were obtained using the RC1 reaction coordinate values in the range from −4.7 Å to −2.6 Å and the dimensionless RC2 reaction coordinate values ranging from 0.3 to 0.8, in simulating the forward reactions of the two reaction steps of the simulated mechanism. When the mid-plane reaction coordinate, RC2 has a value smaller than 0.5 (RC2 <0.5), the hydrogen is still bound to the C7 atom of the Wheland intermediate, and when RC2 equals 0.5 (RC2 = 0.5), the hydrogen is halfway between the carbon of the Wheland intermediate, and the oxygen of E346.

## Results and Discussion

### Potential Energy Reaction Paths

The starting structure for QM/MM potential energy adiabatic mapping calculations was the enzyme-substrate (ES) complex/reactant complex (RC) structure, minimized at the B3LYP/6–31+G(d)-CHARMM27 level. The calculated potential energy barrier for the reaction of the Wheland intermediate formation was 4.0 kcal/mol (Figure S2) and the reaction was exothermic with reaction energy of −8.6 kcal/mol.

**Figure 5:**
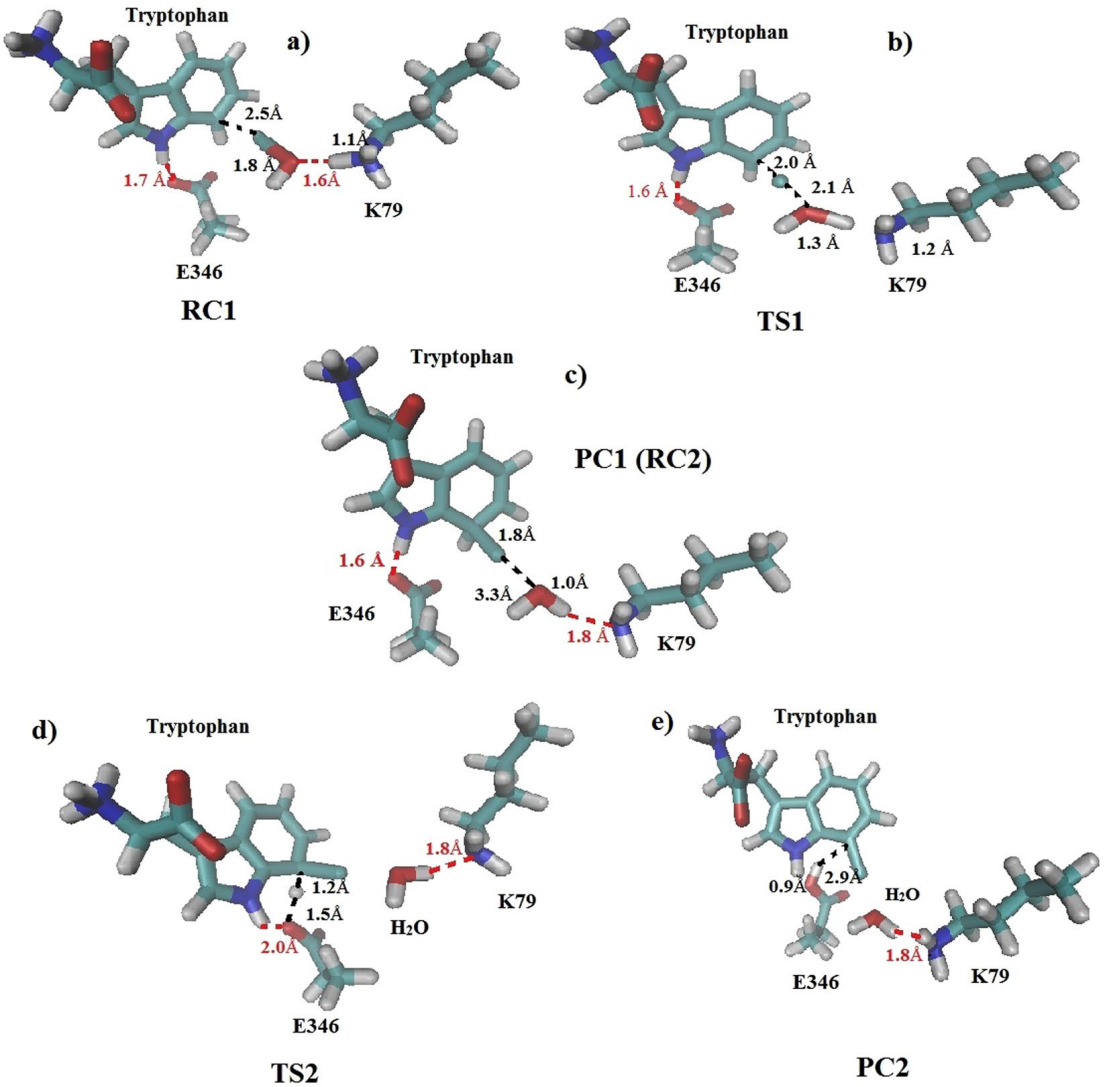
(**a**) Reactant complex (RC1) structure, (**b**) Transition state (TS1) structure, and (**c**) Product complex (PC1) structure from the potential energy scan of the first step of the reaction mechanism - the Wheland intermediate formation. Transition state (TS2) structure and (**e**) Product complex (PC2) structure from the potential energy scan of the second step of the reaction mechanism – deprotonation of the Wheland intermediate. Key active site interatomic interactions between the reactive species - K79, E346, HOCl and tryptophan’s C7 atom are represented as black dashed lines and hydrogen bonds are represented as red dashed lines.

Two hydrogen bonds were observed in the optimized reactant complex structure (RC1) for the first step of the simulated reaction. The first one, between the K79 hydrogen and the HOCl oxygen (distance 1.6 Å) (Table 1, Fig. 5a), supports the experimentally proposed role of K79 in activating HOCl, for the consequent electrophilic aromatic chlorination of tryptophan^4^. The second one is between the hydrogen atom of the NH group of the indole ring of tryptophan, and the oxygen of the carboxyl group of E346 (distance 1.7 Å). In addition, the HOCl chlorine atom is well positioned for making an electrophilic attack on the 7^th^ carbon of the indole ring of tryptophan (distance 2.5 Å). Indeed, an additional hydrogen bond between HOCl and the oxygen of the E346 peptide bond (out of the quantum region and therefore not shown in Fig. 5) at distance of 1.7 Å helps for this positioning. The last two hydrogen bonds were also found in the transition state (TS1) and the product complex (PC1) structures for the first simulated step of the reaction. An additional hydrogen bond was found in the PC1 structure (Fig. 5c), where it stabilizes a water molecule.

**Table 1:** Active site interactions (Å/degrees) in the RC, TS and PC structures for the different reactive species – tryptophan, HOCl, E346 and K79, from the potential energy reaction paths scans, for the two reaction steps of chlorination of tryptophan.

Bond/Angle (Å/degrees)	Reactant Complex Structure	Transition State Structure	Product Complex Structure (Wheland Intermediate)
*Wheland Intermediate Formation Reaction Step*			
Reaction Coordinate, RC1	−0.7	0.1	1.5
K79 (H-O) HOCl	1.6	1.3	1.0
HOCl (Cl-C) Tryptophan	2.5	2.0	1.8
HOCl (O-Cl)	1.8	2.1	3.3
K79 (N-H)	1.1	1.2	1.8
*Wheland Intermediate Deprotonation Reaction Step*			
Reaction Coordinate, RC2	−1.7	−0.3	2.0
Tryptophan (H-O) E346	2.9	1.5	0.9
Tryptophan (C-H)	1.1	1.2	2.9
Tryptophan (C-H-O) E346	113.4	141.8	127.6

The transition state structure (TS1), shows advancement in the bond formation between HOCl chlorine and the 7^th^ carbon atom of tryptophan, and the bond formation between the hydrogen atom of K79 and the oxygen of HOCl (Table 1, Fig. 5b) that finally leads to formation of 7-chlorotryptophan and a water molecule. The product complex structure (PC1) had both the Wheland intermediate, and the water molecule completely formed (Table 1, Fig. 5c). In the Wheland intermediate structure, the bond between the chlorine and the carbon atom at 7^th^ position in the indole ring of tryptophan is formed, and the aromatic hydrogen at position 7 of the indole ring, points toward E346. This atomic arrangement is consistent with the reaction of deprotonation of the Wheland intermediate, performed by the carboxylate group of E346.

Deprotonation of the Wheland intermediate is the second step in the regiospecific chlorination of tryptophan, leading to formation of the final product 7-chlorotryptophan. An oxygen atom from the carboxyl group of E346 is thought to abstract the proton^4^. The structure of the Wheland intermediate (PC1 in the first step of the reaction mechanism) was used as a starting structure for the reaction modelling of the second step (Fig. 5c). The activation energy barrier for deprotonation of the Wheland intermediate by the oxygen atom from the carboxyl group of E346 is 9.4 kcal/mol and the reaction energy is −24.3 kcal/mol. In the transition state structure (TS2), the bond between the tryptophan hydrogen atom and the E346 oxygen is partially created and in the product complex structure (PC2), this bond is completely formed (Table 1, Fig. 5e).

### Free Energy Reaction Paths

**Figure 6:**
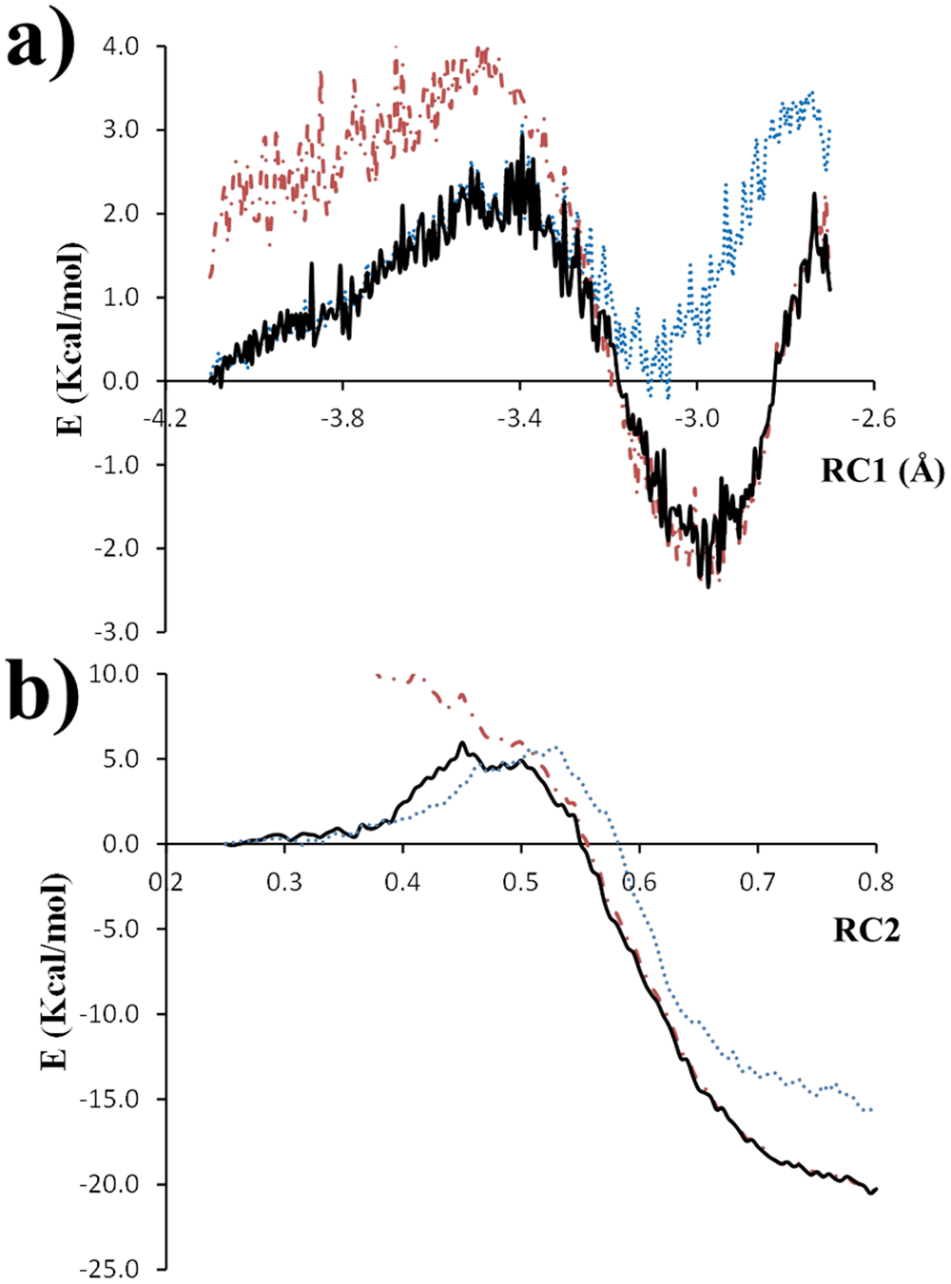
QM/MM-MD free energy reaction profiles (**a**) Wheland intermediate formation and (**b**) deprotonation of the Wheland intermediate. Unidirectional Hummer and Szabo’s estimator applied to the forward pullings (brown line) and to the reverse pullings (blue line). Bidirectional Minh and Adib estimator - shown as a black line.

The calculated free energy reaction paths (Potential of Mean Force, PMF) for both reaction steps of the enzymatic chlorination of tryptophan, are shown in Fig. 6. The bidirectional PMF estimator of Minh-Adib (Equation 2) was used to reconstruct the unbiased PMF along the two RCs and to optimally combine the forward and backward profiles thus, accelerating the convergence of the free energy.

The PMF using unidirectional Hummer-Szabo estimator is getting more biased, as the states are further away from the starting equilibrium state, showing a clear overestimation of PMF. Obtaining PMF, using MSMD methodology at DFT level, requires high pulling velocity due to its large computational cost. This leads to growing error with increasing the distance from its original equilibrium state. It has been shown that bidirectional PMF estimators improve resultant PMF profiles by estimating the optimal free energy difference using data from forward and backward pulling^37^. Therefore, the free energy differences between reactants, transition states and product complexes were derived from Minh-Adib estimator profile, and do not present the biasing on the PMF profile, because of the use of forward and backwards reaction profiles (Table 2).

**Table 2:** Free energies differences (∆G, kcal/mol) from multiple steered molecular dynamics (MSMD) simulations for the two steps of chlorination of tryptophan. Values derived from Bidirectional Minh-Adib estimator. Standard errors of the mean values are shown.

Reaction Step of chlorination of tryptophan	Reaction Free Energy, ΔG	Activation Free Energy for the forward reaction ΔG^‡^	Activation Free Energy for the backward reaction ΔG^‡^
First step (RC1)	−2.4 ± 0.7	3.0 ± 0.9	5.4 ± 0.9
Second step(RC2)	−20.4 ± 0.2	6.0 ± 1.0	26.5 ± 1.0

**Figure 7:**
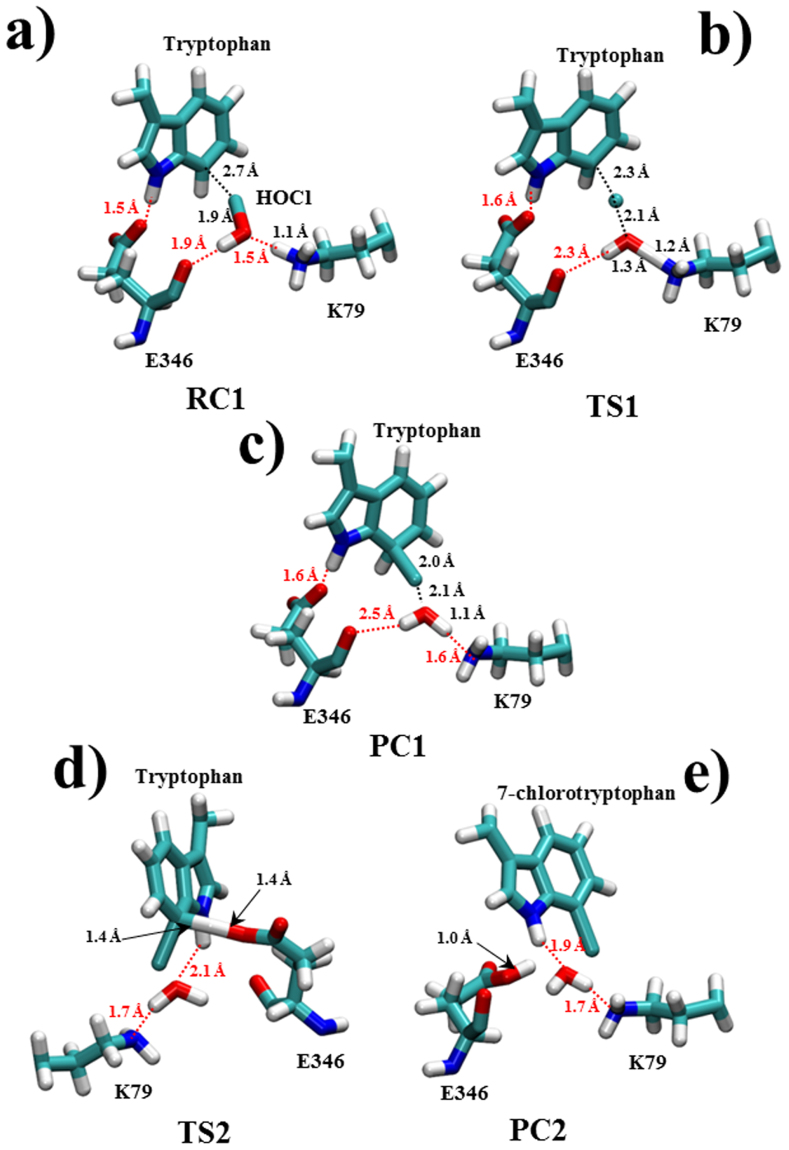
Averaged structures from the free energy scans of (**a**) reactant complex (RC1), (**b**) transition state (TS1) and (**c**) product complex (PC1) for the first step of the reaction mechanism - Wheland intermediate formation and averaged structures of the free energy scans of (**d**) transition state (TS2) and (**e**) product complex (PC2) for the second step of the reaction mechanism – Wheland intermediate deprotonation. Key active site interatomic interactions between the reactive species - K79, E346, HOCl and tryptophan’s C7 atom are given as black dashed lines and hydrogen bonds are represented as red dashed lines.

The free energy barrier for the first reaction step is about 3.0 kcal/mol, resulting in an exothermic reaction with a free energy of −2.4 kcal/mol. Importantly, in the first reaction step HOCl is activated for electrophilic attack by two strong hydrogen bonds with K79 and E346 residues. The transition state structure keeps a weaker hydrogen bond with E346 and forms a new strong hydrogen bond with K79 that remains in the product complex structure thus, helping to stabilize both the transition state and final product complex structures (Table 3 Fig. 7).

**Table 3:** Active site interactions (Å/degrees) in the RC, TS and PC structures (Å/degrees) for the different reactive species – tryptophan, HOCl, E346 and K79, from the free energy reaction paths scans for the two reaction steps of chlorination of tryptophan, (±standard deviation).

Bond/Angle (Å/degrees)	Reactant Complex Structure	Transition State Structure	Product Complex Structure
*Wheland Intermediate Formation Reaction Step*			
Reaction Coordinate, RC1	−4.1	−3.4	−3.0
K79 (H-O) HOCl	1.5 ± 0.0	1.3 ± 0.1	1.1 ± 0.0
HOCl (Cl-C) Tryptophan	2.7 ± 0.1	2.3 ± 0.1	2.0 ± 0.0
HOCl (O-Cl)	1.9 ± 0.1	2.1 ± 0.1	2.4 ± 0.1
E346 (O-H) HOCl	1.9 ± 0.2	2.3 ± 0.4	2.5 ± 0.5
K79 (N-H)	1.1 ± 0.1	1.2 ± 0.1	1.6 ± 0.1
*Wheland Intermediate Deprotonation Reaction Step*			
Reaction Coordinate, RC2	0.3	0.5	0.8
Tryptophan (H-O) E346	2.5 ± 0.2	1.4 ± 0.1	1.0 ± 0.1
Tryptophan (C-H)	1.1 ± 0.1	1.4 ± 0.1	3.2 ± 0.3
E346 (O-C)	1.3 ± 0.1	1.3 ± 0.1	1.4 ± 0.1
Tryptophan (C-H-O) E346	130 ± 9	156 ± 7	135 ± 13

The calculated free energy path for the second step of the reaction mechanism – the reaction of deprotonation of the Wheland intermediate by the E346 oxygen, shows an activation energy barrier of 6.0 kcal/mol, and reaction energy of −20.4 kcal/mol. Thus, the second reaction step is the rate-limiting one, which is in agreement with the potential energy path calculations (9.4 kcal/mol). The free reaction energy of −20.4 kcal/mol is in agreement with the potential reaction energy of −24.3 kcal/mol and suggests about an exothermic reaction. The reaction free energy for the whole reaction of chlorination of tryptophan is −22.8 kcal/mol.

### Structure and Charge Analysis of Reaction Complexes

**Figure 8:**
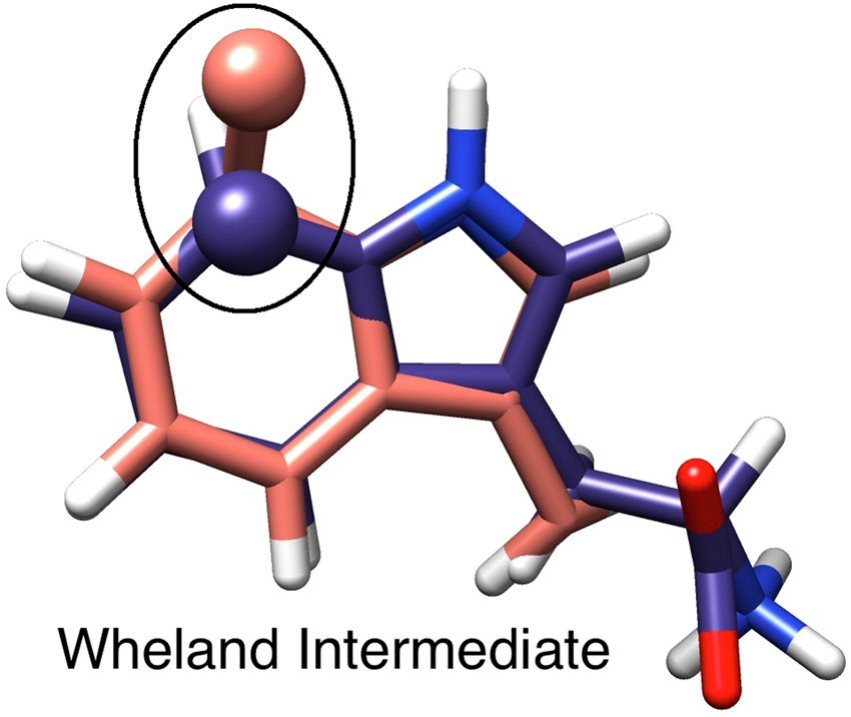
Superimposed QM/MM optimized Structures of the Wheland intermediate, potential energy profile one (purple) and free energy profile one (orange). The 7^th^ carbon atom of the indole ring of tryptophan, chlorinated and bound to its hydrogen atom, is highlighted in a black circle.

Key interactions, corresponding to reactants, transition states, and products for both reaction steps of the mechanism, averaged through several snapshots of the hybrid QM/MM-MD, are presented in Table 3, Fig. 7. For example, there is a strong hydrogen bond between the K79 hydrogen and the oxygen of HOCl (1.5 Å), in the reactant complex (RC1) for the reaction of formation of the Wheland intermediate. Indeed, this hydrogen bond facilitates the process of chlorine transfer from HOCl to tryptophan, and the formation of a water molecule. An additional hydrogen bond of 1.9 Å between HOCl and the carboxyl group of E346 is found to stabilize the reactive complex, and is found to disappear after the first reaction step. A similar hydrogen bonding network, stabilizing the RC, TS, and PC structures was also found in the potential energy scans. The superimposed structures of the Wheland intermediate from the potential energy and free energy scans are very similar and are shown in Fig. 8.

**Figure 9:**
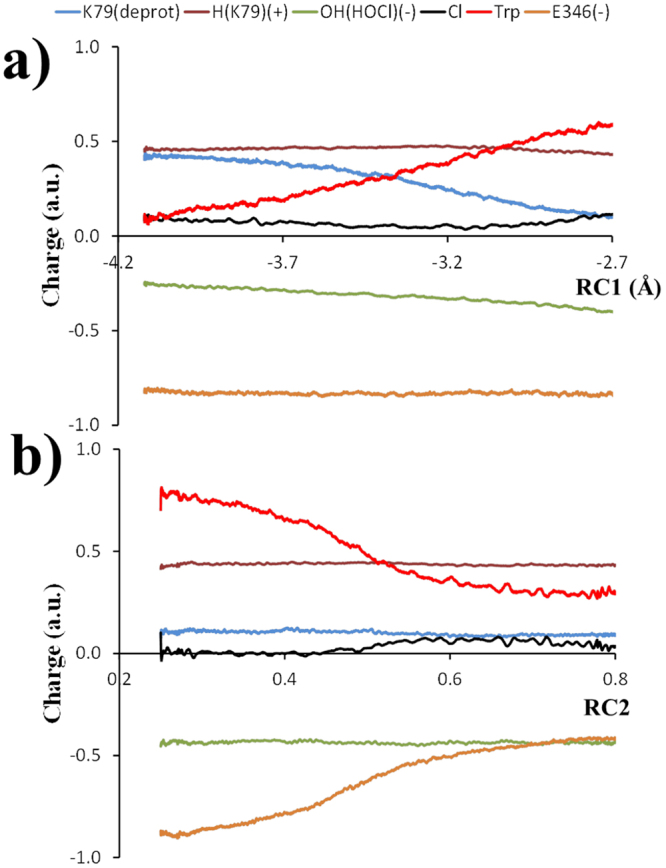
Average Muliken charges for the fragments along (**a**) RC1 (Å) and (**b**) RC2 (dimensionless). K79 deprotonated (blue line), H (K79) (+) (brown line), Cl (black line), tryptophan (red line), E346 (−) (orange line). OH^−^ (HOCl) (−) green line.

These interactions facilitate the electrophilic attack of Cl to the C atom at 7^th^ position of the indole ring of tryptophan, with an observed charge redistribution/reorganization of ~0.4 a.u. (Fig. 9). The Cl atom is a subject of a major atomic displacement between the chlorine and the C atom at the 7^th^ position of the indole ring of tryptophan (RC1, Fig. 6) along bond formation, starting from 2.7 Å in the reactant complex, going up to 2.0 Å in the product complex, and located at 2.3 Å in the transition state structure (Table 3). Moreover, minor displacements of 0.4 and 0.5 Å are observed for the HOCl (O-Cl) bond breaking, and HOCl-K79 (O-H) bond formation, respectively.

The electrophilic attack of Cl to tryptophan, leads to the formation of a positively charged Wheland intermediate, stabilized by electrostatic interaction with the carboxylate group of E346. In addition, there is a strong hydrogen bond between the E346 carboxylate oxygen, and the hydrogen from the indole NH group of the tryptophan, with maximum distance probability d O-H = 1.6 Å, which represents a QM/MM-MD trajectory occupation of 88% (the percentage of structures exhibiting the particular hydrogen bond). The close proximity of the hydrogen atom of the Wheland intermediate to one of the oxygen atoms from the carboxyl group of E346 allows for its quick and easy transfer. The transition state is an intermediate structure, where the hydrogen atom of the Wheland intermediate is located at 1.4 Å from the carboxyl group oxygen of E346 with an almost planar angle of 156° between the C-H-O atoms. In the product complex structure, the proton is entirely transferred to the carboxyl group oxygen of E346 (d_O-H_ = 1.0 Å).

In order to study charge redistribution amongst the main chemical species participating in the two reaction steps of the simulated reaction mechanism, a few species/fragments, that are part of the quantum region (QM) of the simulated system, were defined (Fig. 9) as follows: deprotonated K79 residue (K79(deprotonated)), a proton from the protonated amino group (NH_3_^+^) of K79 (H(K79)^+^), hydroxyl group (OH^−^) of HOCl (OH(HOCl)), chlorine atom (Cl) of HOCl, tryptophan (Trp), and deprotonated E346 residue (E346(^−^)), respectively. The charge on these fragments was obtained by adding the Mulliken atomic charges at each fragment for each trajectory snapshot. The global charges for each fragment, of the two reaction steps, as a function of the RC, and averaged from all forward pullings are given in Fig. 9. As RC1 increases, there is a slight polarization, in direction of tryptophan, due to charge transfer mainly from K79 deprotonation, as seen by the decrease in the positive charge from K79 (deprotonated) fragment, and the corresponding charge increase on the tryptophan fragment. Similarly, as RC2 increases, there is a slight positive charge transfer (~0.4 a.u.) from the tryptophan fragment to E346 fragment. Thus, there is a net redistribution of ~0.4 a.u of positive charge transferred from K79 to E346 during the whole process of chlorination of tryptophan. The K79 fragment is the one that is the most involved in the charge transfer to tryptophan (with about 0.4 a.u.), but there are other contributions too. HOCl molecule and in particular its OH fragment, also contribute a little to the charge transfer (with about 0.1 a.u.).

### Similarities and Differences between the Potential Energy and Free Energy Reaction Paths

Comparison between the activation and the reaction energies of the two steps of the simulated reaction mechanism calculated at the different levels of theory was performed.

There is a very close agreement between the activation energies calculated for the potential energy path and the free energy path for the first step of the simulated mechanism - the reaction of Wheland intermediate formation within 1 kcal/mol (Table 4). A difference of about 6 kcal/mol for the reaction energy for the same reaction was found between the free energy and the potential energy pathways, with the potential energy path providing a more exothermic reaction. This could be attributed to the formation of a higher energy Wheland intermediate in the free energy reaction path simulation. In the free energy PC1 structure, a water molecule is found to participate in a bridge interaction between the chlorine atom of the Wheland intermediate and the nitrogen atom of the amino group of K79. In fact, a more elongated C-Cl bond (d_C-Cl_ = 2.0 Å), and a stronger hydrogen bond between a water molecule and K79 amino group (d_N-H_ = 1.6 Å, Table 3, Fig. 7c) were observed, in respect to the same structure in the potential energy path where the bond distances are 1.8 Å and 1.8 Å, respectively (Table 1, Fig. 5).

**Table 4:** Activation and reaction energies for the formation of the Wheland intermediate, derived from potential energy path and free energy path calculations. Standard errors of the mean values are shown.

Level of Theory	∆E^‡^	∆E
*Potential Energy Path (ΔU) for Wheland intermediate formation*		
B3LYP/6–31+G(d)-CHARMM27	4.0	−8.6
*Free Energy Path (∆G) for Wheland intermediate formation*		
B3LYP/6–31 G/PUPIL-AMBER	3.0 ± 0.9	−2.4 ± 0.7

The calculated activation and reaction energies, for both the potential energy and the free energy path calculations, for the proton abstraction of the Wheland intermediate reaction step performed by the oxygen atom of E346, are presented in Table 5.

**Table 5:** Activation energy and reaction energy for the reaction of deprotonation of the Wheland intermediate by E346 oxygen atom derived from potential energy path and free energy path calculations. Standard errors of the mean values are shown.

Level of Theory	∆E^‡^	∆E
*Potential Energy Path (∆U) for Wheland intermediate deprotonation*		
B3LYP/6–31+G(d)//CHARMM27	9.4	−24.3
*Free Energy Path (∆G) for Wheland intermediate deprotonation*		
B3LYP/6–31 G/PUPIL-AMBER	6.0 ± 1.0	−20.4 ± 0.2

The calculated activation energy of the deprotonation reaction from the free energy calculation is lower by 3.4 kcal/mol than the one calculated from the potential energy scan. The free energy simulations indicate less exothermic reactions, as compared to the potential energy path. These energetic alterations can be due either to methodological differences between the two and/or due to entropic contribution. Despite the small difference in the basis set used for the QM subsystem between potential energy path and free energy path simulations, there are important methodological differences such as the applied periodic boundary conditions, contribution of the conformational entropy, and the fact that the full protein system is taken into account, that are considered only in the free energy path simulations. In the QM/MM potential energy scans the solvated system was truncated at 25 Å from the CZ atom of tryptophan whilst, in the free energy scans a larger system (the whole protein in a solvent box) was used. Such differences could be an origin of some deviations in the results.

**Figure 10:**
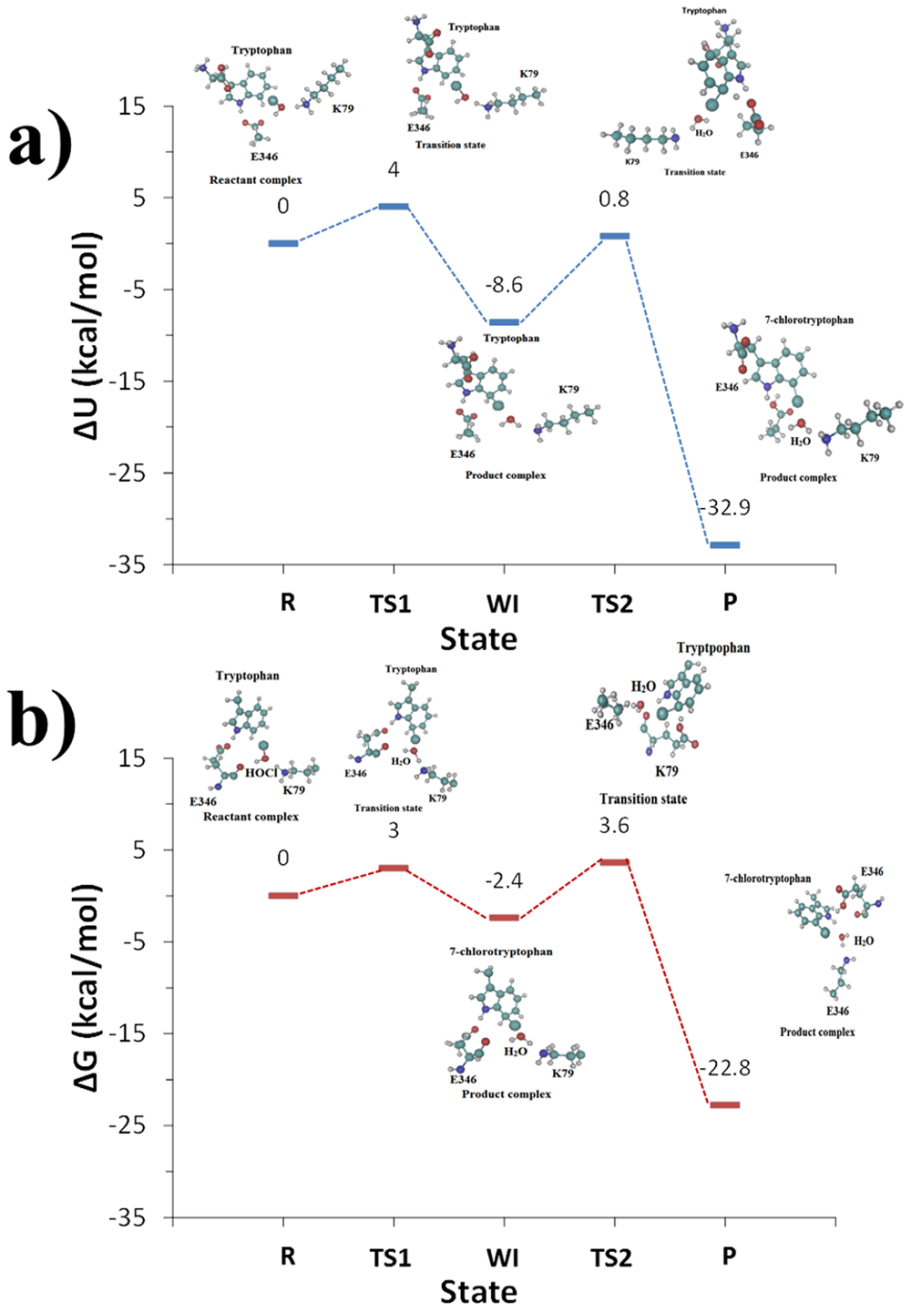
(**a**) Potential energy and (**b**) free energy diagrams derived from potential energy and free energy path profiles at *ab initio* level, respectively. The stationary states/structures of RC, TS and PCs are given for the two steps of the overall reaction mechanism of chlorination of tryptophan.

The potential energy surface and the free energy surface for the simulated reaction mechanism of chlorination of tryptophan are given in Fig. 10.

Both the potential energy and free energy calculations show that the rate-limiting step appears to be the second one – the proton abstraction (Tables 4 and 5). Entropy considerations and methodology differences lead to a reduction of the energy barrier and also to a higher energy Wheland intermediate state. In fact, the introduction of kinetic energy in the two simulation steps of the calculated reaction pathways leads to a substantially more unstable arenium cation complex with a lower activation energy, that facilitates the final proton abstraction step when compared to the potential energy path which always finds/favors the most stable conformation. As a consequence, the overall reaction free energy is −22.8 kcal/mol and the global PMF maximum (the activation energy) is 3.6 kcal/mol, calculated in respect to the initial reactant complex energy.

In this first computational study of the reaction mechanism of tryptophan 7-halogenase we applied two different computational QM/MM strategies with DFT treatment of the QM part to explore the feasibility of a proposed catalytic mechanism of chlorination of tryptophan, to characterize structures of the stationary points (reactant complexes, transition states, product complexes) and to determine the energetics of the process. Despite all the differences in the two QM/MM methodologies, the structures of the stationary points and the observed hydrogen bonding networks characterized in the two methods are very similar. The rate-limiting step appears to be the same - the deprotonation of the Wheland intermediate. The activation barriers for the two reaction steps are also similar. The information from the potential energy surface with minima energy structures was complemented by the dynamic information that is derived through the free energy simulations. The provided atomistic and electronic structural insights in the reaction mechanism of enzymatic tryptophan chlorination would contribute for molecular design of halogenase enzymes with desired activity and regioselective properties for production of biologically and pharmaceutically active natural products that contain a chlorine atom as well as for further implementation of enzymes in the synthesis of chlorinated organic compounds.

## Conclusions

The enzymatic mechanism of regioselective aromatic electrophilic chlorination of tryptophan by hypochlorous acid in tryptophan 7-halogenase was investigated for first time using two different QM/MM potential energy and QM/MM-MD free energy methodologies at DFT level of theory for the QM parts. The stationary points (reactant complexes, transition states and product complexes) along the reaction paths were characterized with emphasis on the most important interactions. For the Wheland intermediate formation the potential energy and free energy path calculations showed nearly the same energy barriers, however, the free energy path profile yields a higher energetic state of the Wheland intermediate. Both methods, however, provide similar hydrogen bonding interactions in the active site that stabilize the stationary points along this reaction step. The calculations for the second step, deprotonation of the Wheland intermediate, revealed that one of the oxygens of the carboxyl group of E346 plays the role of proton acceptor, while the other oxygen is involved in a hydrogen bond interaction with the nearby NH group of tryptophan. In the reaction of chlorination of tryptophan, a net charge transfer of ~+0.4 a.u, was observed from K79 to E346, and the potential energy paths and free energy paths calculations indicate that the proton abstraction step is the rate-limiting one. Both reaction profiles show a similar global maximum of energy close to ~4 kcal/mol; meanwhile the reaction energy differs with values of −32.9 kcal/mol and −22.8 kcal/mol for the potential energy path and free energy path profiles, respectively. Calculations confirm the experimentally suggested roles of K79 and E346 in activating HOCl, assuring the regioselectivity of the process and provide atomistic insight about their roles. The insights from this first computational study of the reaction mechanism of tryptophan 7-halogenase would be valuable for a design of halogenating enzymes with desired properties with broad applications in chemical biology, pharmaceutical industry and organic synthesis.

## Electronic supplementary material

Supporting Information
