# Supplementary material for: Mechanistic Insights into the Reaction of Chlorination of Tryptophan Catalyzed by Tryptophan 7-Halogenase
Source: Sci Rep. 2017 Dec 12;7:17395. doi: 10.1038/s41598-017-17789-x (PMC5727139; doi:10.1038/s41598-017-17789-x)
Supplement: Supplementary file 1 — Supporting Information [file 41598_2017_17789_MOESM1_ESM.pdf]

Supplementary Information

Mechanistic Insights into the Reaction of Chlorination of Tryptophan Catalyzed by Tryptophan 7-Halogenase

Tatyana G Karabencheva-Christova,[1,3,*] *Juan Torras,*[2,*] *Adrian J Mulholland,*[3] *Alessio Lodola,*[4] *and Christo Z Christov,*[1,3]

[1] Department of Chemistry, Michigan Technological University, Houghton, 49931, MI, USA

[2] Department of Chemical Engineering, Escola d'Enginyeria de Barcelona Est (EEBE), Universitat Politècnica de Catalunya, C. Eduard Maristany 10-14, 08019 Barcelona, Spain

[3] Centre for Computational Chemistry, School of Chemistry, University of Bristol, Cantock's Close, Bristol, BS8 1TS, UK

[4] Pharmacy Department, Università di Parma, V. le P.G Usberti 27/A, Campus Universitario, 431124 Parma, Italy

Corresponding authors: tatyanak@mtu.edu; joan.torras@upc.edu

System set up

System preparation for Potential Energy Path simulations

The X-ray crystallographic structure with PDB ID: 2AR8 [1] was used for modelling. It contains a chloride ion (Cl^-), a flavin adenine dinucleotide (FAD) cofactor and the reaction product: 7-chlorotryptophan, which was modified to the substrate – tryptophan. In addition, an OH group was added to the chloride ion (Cl^-) in order to model the hypochlorous acid.

The chemical reaction of interest is localized to the active site of the enzyme. Therefore, in order to reduce the computational time required for simulations, stochastic boundary molecular dynamics (SBMD) simulations were applied [2]. The system was divided into two regions. A "reaction" region that includes all atoms within 21 Å from the centre of the sphere - the 7th carbon atom from the indole ring of tryptophan - and a "buffer" region for all atoms that are between 21 Å and 25 Å away from the centre. Atoms in the reaction region were free to move according to Newton's second law whilst, the non-solvent heavy atoms in the buffer region were harmonically restrained with force constants ranging from a maximum value at 25 Å to zero at 21 Å from the reaction centre. The restraints acting on the buffer region were necessary because the protein was truncated. Since part of the protein had been removed the remaining part might thus become unstable during molecular dynamics simulations. Therefore, the harmonic restraints helped to preserve the correct structure of the system. A deformable boundary potential was applied to the water oxygen atoms (restraining the water molecules within the sphere). The SHAKE algorithm [3] was applied to fix the length of all MM bonds involving hydrogen atoms and a 1 fs time step was used. Glutamate and aspartate residues in the buffer region were protonated to neutralize the negative charge. To obtain a neutral net charge for lysine and arginine residues in the buffer region, patch residues of the

same geometry, but with scaled partial atomic charges were used. After equilibration at 300 K of all water molecules keeping all the other atoms fixed, the water molecules were optimized by 2000 steps of steepest descent minimization and 5000 steps of adopted basis Newton-Raphson (ABNR) energy minimization. Subsequently, the whole system was equilibrated using SBMD. The time step was 0.5 fs. The simulation was divided into two phases: a heating phase of 30 ps, needed to increase the temperature from 0 to 300 K and a production phase of 250 ps. After that, the structure was minimized by two sets of 1000 steps of SD and 5000 steps of ABNR. The system contains 8197 protein atoms, 87 FAD atoms, 3 HOCl atoms, 27 tryptophan atoms and 678 crystal water atoms (226 water molecules). In total, there were 8992 atoms. The missing hydrogen atoms in the crystal structure were added according to the protonation states of the ionogenic groups with the HBUILD routine [4], available in the CHARMM27b2 package [5]. The protonation states (pK_a) of all histidine residues were determined according to their local environment and close contacts with the help of the WHATIF program (http://swift.cmbi.kun.nl/whatif [6]). Subsequently, all hydrogen atoms were optimized by molecular mechanics minimizations of 500 steps of steepest descent (SD) and 1500 steps of adopted basis Newton Raphson (ABNR) using CHARMM program, version 27b2 [5]. The system was then solvated by superimposing a 60 Å edge box of 8000 previously equilibrated TIP3P [6] water molecules and deleting all waters whose oxygen atoms lie within 2.6 Å distance of another heavy atom. Afterwards, the system was optimized. Subsequently, the system was truncated by deleting each protein residue and each water molecule without at least one heavy atom within 25 Å of the reaction centre (the 7th carbon atom of the indole ring of the substrate tryptophan). Neutralization of the system was then performed, because any charged residues on the surface of the protein may give unrealistically large contributions to the electrostatic component of the energy. The charged residues on the protein surface were neutralized by patching them with residues of exactly the

same geometry, but with neutral net charge. The truncated system contains 7582 atoms from which there are 5857 protein atoms and 1607 water atoms (536 water molecules).

QM/MM set-up

The system was divided into QM and MM parts. The quantum chemical region of the system contained 57 atoms in total and had a net charge of zero. In order to treat the covalent bonds crossing the QM/MM boundary link atoms were added. Since the link atoms were included in the QM Hamiltonian they were exposed to polarisation from the MM point charges. In order to counteract this polarisation and to avoid any non-physical effects the charges on the MM atoms replaced by the link atom were set to zero. And the charges on the atoms adjacent to the link atoms were modified to retain a neutral charge at the QM/MM interface. No cut-offs for non-bonded interactions were used. MM energies and forces were calculated with the Tinker package.

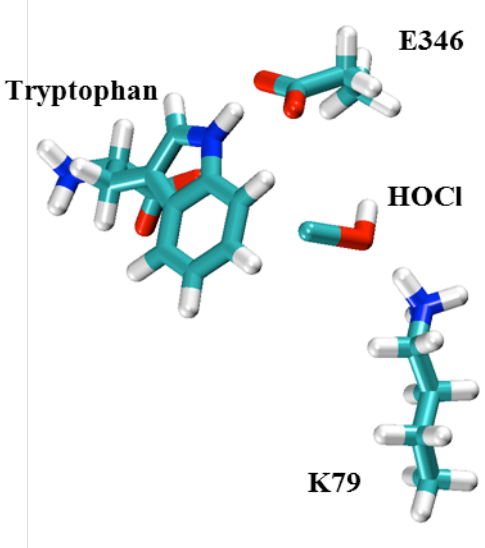

Figure S1 Representation of the quantum mechanical (QM) region of the reactant complex (RC) used for potential energy reaction path calculations. It includes the substrate - tryptophan, the chlorinating agent - HOCl and the side chains of E346 and K79.

Potential Energy Reaction Path Modelling

The reactant and product complex structures were minimized without restraints in order to verify their stability. The transition states were determined approximately as the highest energy points along the respective reaction profile. During all of the adiabatic mapping calculations atoms which were further than 20 Å away from the tryptophan carbon atom at 7th position of its indole ring, were held fixed.

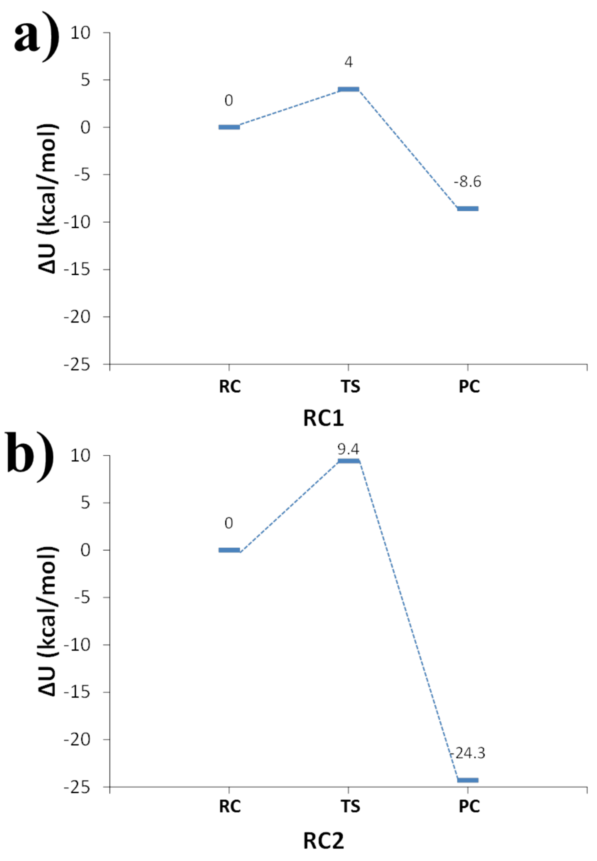

Figure S2 QM/MM adiabatic mapping potential energy reaction profiles of the reaction mechanism of chlorination of tryptophan in two steps: (a) Wheland intermediate formation and (b) Wheland intermediate deprotonation. Reaction coordinates RC1 and RC2 were used and the energy is given in kcal/mol. The positions of the stationary points - RC (reactant complex), TS (transition state) and PC (product complex) on the potential energy surface are presented.

System preparation for Free Energy Path simulations

The QM/MM-MD simulations were performed using the Amber-PUPIL-NWChem interface. The same crystal structure (PDB ID: 2AR8) was used as the one used in the potential energy QM/MM calculations. The protein was solvated under periodic boundary conditions within a parallelepiped box (91.20 x 75.50 x 80.65 Å), containing 18779 TIP3P [7] water molecules. Initially, the box was equilibrated and brought to 298 K by classical MD, using the AMBER 14 program [8]. Specifically, the system was first subject to 2000 minimization steps using MM to remove any clashes, and then was heated to 298 K for 60 ps at constant volume. A time step of 2 fs and Langevin thermostat with 2.0 ps^{-1} collision frequency were used. Finally, the system was relaxed for 0.5 ns at constant pressure (1atm) and pressure relaxation time of 1 ps using MM. For all these steps, the SHAKE algorithm was used to restrain bonds containing hydrogens. At this point, the system was considered sufficiently equilibrated and another 5 ns MD run at constant volume was used to extract 15 uncorrelated structures. These 15 structures were used as input structures for further trajectories in generating the free energy profiles. Next, each of these initial structures was relaxed within the hybrid QM/MM-MD framework during 0.5 ps (1000 steps) at the B3LYP/6-31G level. Distance constraints were applied to HOCl, K79 and tryptophan in order to keep the geometry of the QM region close to the initial structure.

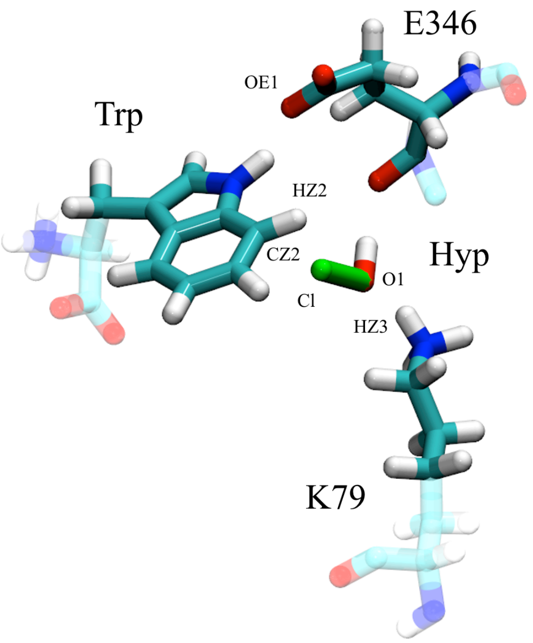

Figure S3 Representation of the quantum mechanical (QM) region used in the hybrid free energy QM/MM-MD simulations. HOCl, tryptophan, K79 and E346 moieties that are part of the QM region are highlighted, and their parts that belong to the classical Molecular Mechanical (MM) region are represented as glassy particles.

References

1. Dong, J.C., Flecks, S., Unversucht, S., Haupt, C., van Pee H.K. & Naismith, H.J. *Science,* **309**, 2216-2219 (2005).

2. Brooks, L.C. & Karplus, M. *J. Mol. Biol.,* (**208**, 159-1811989).

3. van Gunsteren, F. W. & Berendsen, C. J. H. *Mol. Phys.,* **34,** 1311-1327 (1977).

4. Brünger, T. A. & Karplus, M. *Proteins: Str., Funct., Genet.,* **4**, 148-156 (1988).

5. Brooks, R. B. , Bruccoleri, E. R., Olafson, D. B., States J. D. & Swaminathan, S. *J. Comp. Chem.,* **4**, 187-217 (1983).

6. Rodriguez, R., Chinea, G., Lopez, L., Pons, T. & Vriend,G. *Bioinformatics,* **14**, 523-528 (1998).

7. Jorgensen, L. W., Chandrasekhar, J. , Madura, D.J., Impey W. R. & Klein, L. M. *J. Chem. Phys.,* **79**, 926-935 (1983).

8. D. A. Case, V. Babin, J. T. Berryman, R. M. Betz, Q. Cai, D. S. Cerutti, III, T. A.Darden, R. E. Duke, H. Gohlke, A. W. Goetz, S. Gusarov, N. Homeyer, P. Janowski, J. Kaus, I. Kolossváry, A. Kovalenko, T. S. Lee, S. Le Grand, T. Luchko, R. Luo, B. Madej, K. M. Merz, F. Paesani, D. R. Roe, A. Roitberg, C.Sagui, R. Salomon-Ferrer, G. Seabra, C. L. Simmerling, W. Smith, J. Swails, R. C. Walker, J. Wang, R. M. Wolf, X. Wu and P. A. Kollman, *AMBER 14*, University of California: San Francisco, 2014.
